# Examining the Link Between Public Transit Use and Active Commuting

**DOI:** 10.3390/ijerph120404256

**Published:** 2015-04-17

**Authors:** Melissa Bopp, Vikash V. Gayah, Matthew E. Campbell

**Affiliations:** 1 Department of Kinesiology, The Pennsylvania State University, University Park, PA 16802, USA; E-Mail: mec378@drexel.edu; 2 Department of Civil and Environmental Engineering, The Pennsylvania State University, University Park, PA 16802, USA; E-Mail: gayah@engr.psu.edu

**Keywords:** active transportation, public transit, physical activity, health, commuting

## Abstract

*Background*: An established relationship exists between public transportation (PT) use and physical activity. However, there is limited literature that examines the link between PT use and active commuting (AC) behavior. This study examines this link to determine if PT users commute more by active modes. *Methods*: A volunteer, convenience sample of adults (*n* = 748) completed an online survey about AC/PT patterns, demographic, psychosocial, community and environmental factors. *t*-test compared differences between PT riders and non-PT riders. Binary logistic regression analyses examined the effect of multiple factors on AC and a full logistic regression model was conducted to examine AC. *Results*: Non-PT riders (*n* = 596) reported less AC than PT riders. There were several significant relationships with AC for demographic, interpersonal, worksite, community and environmental factors when considering PT use. The logistic multivariate analysis for included age, number of children and perceived distance to work as negative predictors and PT use, feelings of bad weather and lack of on-street bike lanes as a barrier to AC, perceived behavioral control and spouse AC were positive predictors. *Conclusions*: This study revealed the complex relationship between AC and PT use. Further research should investigate how AC and public transit use are related.

## 1. Introduction

Participation in regular physical activity has been associated with a number of benefits including a reduction in morbidity and mortality from cardiovascular disease, obesity, diabetes, certain cancers, and mental health disorders [1]. Despite these known benefits, participation in regular physical activity has been declining [2], and national self-report data indicates that less than 50% of the population currently meets recommendations for physical activity [3,4]. This is a potential contributing factor to growing rates of obesity and metabolic related disorders in the United States [5]. Since physical activity is so vital to a healthy population, it is important to consider all the possible domains of physical activity—Including leisure-time (discretionary or recreational), occupational (work-related), domestic (housework/yard work), and transportation (walking or biking for travel)—To identify which areas can be improved [6]. The majority of the current literature on health outcomes refers primarily to leisure-time activity [1]; however, with sub-optimal rates of participation, an examination of all types of activity is warranted to address population-level health.

The level of physical activity involved in transportation is of particular interest because most people must travel daily. Performing some or all of this travel on active modes—Such as walking or bicycling—Can lead to daily physical activity, which can have significant health benefits. For example, data from national surveys has found negative relationships between active travel and self-reported obesity [7,8,9], though it should be noted that other evidence suggests the relationship is not as well established [10]. Other documented health benefits of active commuting (AC) include a reduced risk of cardiovascular disease and all-cause mortality [11,12,13] and improved mental health [14,15,16]. Active commuting has also been identified as a promising measure to increase physical activity within the population as a whole; e.g., Healthy People 2020 physical activity objectives include initiatives to increase the proportion of active transportation trips as a part of the overarching goal to achieve longevity and health equity [17]. In addition to the physical and mental health benefits, there are also notable ecological/environmental and economic benefits associated with increased AC [18,19,20]. Examples include reduced carbon emissions, reduced fuel consumption and increased participation in the community.

However, travel data suggest that active commuting represents a small proportion of trips. When considering one of the most commonly made trips—The daily trip to work—Active modes are among the least common methods of travel in the United States. While active commuting modes are successful in some smaller, low-population metropolitan areas (e.g., Ithaca, Corvallis, Ames for walking, and Corvallis, Eugene, Fort Collins, Davis for biking) [21], they have been less successful on a national level. Data from the Omnibus Household Survey indicate that the majority of commuters in the US (about 86%) use a personal vehicle to travel to and from work, while only a very small portion of the population either walked or biked to work (3%) [22].

Another travel mode of interest is mass transit (*i.e.*, public transportation—PT), which seems to be on the rise recently, potentially due to number of factors: increased fuel costs, congestion and urbanization in the United States, improved service, changing values. Data from the American Public Transportation Association show that the number of trips made by public transportation has increased by 30% from 1995 to 2010 [23]. More people also use transit to get to work than active travel modes; as per the Omnibus Household Survey, about 5% of work trips nationally are made by transit while only 3% are made by walking or bicycling [22]. Public transit use is particularly high (as much as 12%–30%) in large, high-population metropolitan areas such as New York City, Washington DC, Chicago, Boston and San Francisco. US Census data also indicate that PT use tends to be particularly high among Non-Hispanic Blacks, Asians, Hispanics or those born outside the United States [21]. 

Since PT users typically must walk or bicycle to access their transit stops, it is not surprising that PT use has been linked to increased participation in physical activity [24,25,26,27,28]. Therefore, one would expect public transit users to enjoy significant health benefits. This has been confirmed in the literature. For example, an analysis of the 2001 US National Household Travel Survey showed public transit use was associated with less obesity [29], which was also confirmed in a more recent study [30]. Other notable health benefits of public transit use separate from greater activity include lower passenger fatality rates, lower stress levels and improved air quality [31,32,33]. An Australian study of university students also found that those individuals who used public transit were more likely to achieve the public health goal of 10,000 steps/day measured by pedometer when compared with those students who reported higher private motor vehicle use [34]. Lachapelle and colleagues concurred with these findings with an American sample while noting that, regardless of neighborhood walkability, psychosocial factors could also influence the relationship between public transit and physical activity [35]. The health benefits associated with public transportation can also have significant monetary value; e.g., Stokes and others [36] estimated that the installation of a light rail transit system in Charlotte, NC (USA) would result in a 9-year cumulative public health cost savings of $12.6 million as a result of increased physical activity and reductions in obesity. Using European data, Rabi and de Nazelle [37] also noted significant cost savings and health improvement associated with decreased car travel. 

Despite the established relationship between public transportation and physical activity, there is limited literature that examines the link between public transit use and active commuting behavior. Similar to studies using health behavior theories to examine general physical activity participation, using a theoretical foundation can be helpful for explaining behavior [38]. The current study, building on other studies [39,40,41,42,43,44], uses constructs from the Theory of Planned Behavior [45] (perceived behavioral control, subjective norms) and the Social Cognitive Theory [46] (self-efficacy—an individual’s belief in his/her capacity to perform a particular task) to better understand active commuting behavior. Therefore, one of the purposes of this study is to examine if public transit ridership influences the active commuting behavior of individuals; *i.e.*, are those who use transit more likely to use active modes than their non-transit trips? If true, we then seek to determine how individual, social, worksite community or environmental factors could also influence this relationship. Lastly, the study seeks to determine the most important factors that influence the variance in active [32] commuting behavior when controlling for public transit use, since the latter was found to be a significant predictor of the former. The results of this study will reveal insights into potential strategies or policies that might help to increase active commuting among individuals.

## 2. Materials and Methods

To examine the link between public transit use and active commuting behavior, results from a survey of commuters in the mid-Atlantic region of the United States were used. The following section details this data collection effort, the survey questions, and the methods used to analyze the survey results.

### 2.1. Study Design

This cross sectional survey was delivered online using the Qualtrics software program (Provo, UT, USA) from June–December 2011. The study was approved by the Pennsylvania State University Institutional Review Board.

#### Participants and Recruitment

**Figure 1:**
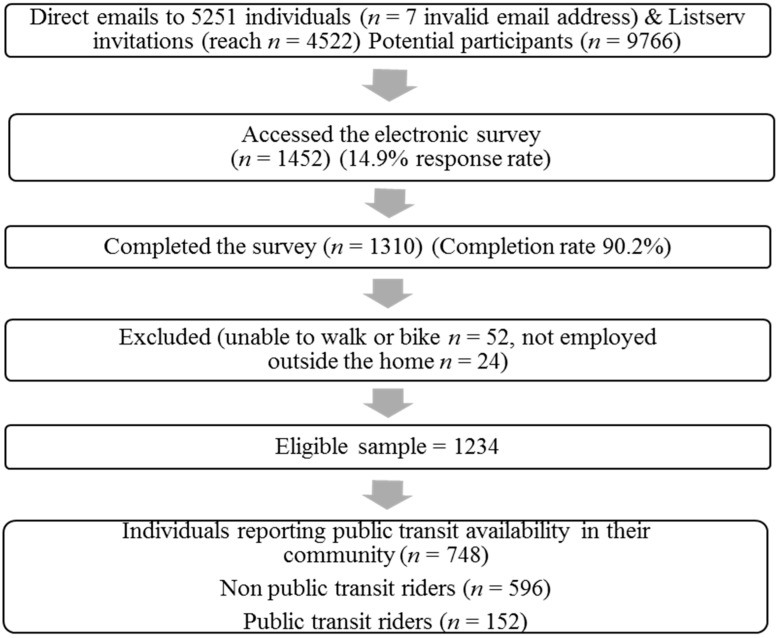
Participant recruitment.

Adults aged 18 years or older, employed full- or part-time outside of the home and physically able to walk or bike were eligible to take part in the survey. Recruitment took place primarily in the mid-Atlantic region of the US (PA, OH, WV, MD, NJ, DE). The websites of large employers (e.g., K-12 school districts, local/county government, private businesses, universities/colleges) in medium-large cities were examined for employee email addresses and individuals were contacted directly via email. These participants received a “*closed*” survey with a unique URL that was only able to be accessed once. When individual employee email addresses were not available, employers were contacted and asked to distribute an electronic invitation to take part in the survey via listserv, e-newsletter, or mass email, with a link to an open URL. In this situation the number of employees receiving the invitation was noted. Among employers contacted to send out the email invitation, only two employers refused to do so, while 84 did not respond to any attempted contacts, and 56 employers sent out the invitation. The e-contact invited people to take part in a “*Commuting survey*”. Participants were asked to self-report if they had access to public transportation in their community, and only those who reported yes were included in the analyses. Recruitment of participants is displayed in Figure 1.

### 2.2. Measures

#### 2.2.1. Commuting Patterns 

Participants were asked to reflect on the previous month and report on average the number of trips per week that they walked, biked, drove in a private vehicle, and took public transportation (where available) to and from work (e.g., an individual reporting walking to and from work would have counted as two trips). The number of individual trips via walking and biking was summed as the total number of active commuting trips. Individuals were dichotomized as either active commuters (>1 AC trips/week) or non-active commuters (0 AC trips/week). Separately, the number of public transit trips was summed and individuals were dichotomized as a public transit riders (>1 trip/week) or non-public transit riders (0 trips/week). This included anyone who used a public transit vehicle, regardless of the mode used to access transit (e.g., walking to the transit stop or driving to a transit stop).

#### 2.2.2. Public Transit Use Patterns

Public transit riders were asked to report how frequently they brought a bicycle on public transit with them and how frequently they got off public transit at an earlier stop for the purpose of walking or biking further to their destination using a 1 (never) to 4 (often) scale. Participants self-reported how far their transit stop was from their home and workplace using a categorical question. Home/work is less than 0.5 miles from the transit stop (coded as 0 miles) and home/work is more than 0.5 miles from the transit stop (coded as 0.5 miles) and a total distance to transit stops was determined (0, 0.5 and 1 miles as possible values). A half mile was used as it would be approximately a 10 min walk, which would be a meaningful bout of physical activity.

#### 2.2.3. Demographics and Medical

Participants reported their age, race/ethnic group, marital status, number of children, education level, income level, sex, and length and type of employment. Participants indicated from a list how many chronic diseases they had and reported their height and weight for body mass index (BMI) calculations. 

#### 2.2.4. Self-Efficacy 

Using a 4-point Likert scale (1 = not at all confident to 4 = very confident with my skills for cycling), participants rated their confidence with their cycling skills in urban areas.

#### 2.2.5. Active Commuting Behavioral Beliefs 

Respondents were presented with eight statements related to physical or mental health benefits of AC (e.g., AC helps me control my weight, can help me to relieve stress) and five statements addressed other AC benefits (e.g., AC is good for the environment, helps me to save money, helps me be more productive at work, makes me a better employee, allows me to focus better). Respondents indicated their agreement with 13 statements about AC using a 7-point Likert scale (1 = completely disagree to 7 = completely agree). A summed score was computed for all 13 items. Based on a previously-tested scale [47] this measure showed excellent reliability (α = 0.91).

#### 2.2.6. Perceived Behavioral Control for active commuting

Using a 7-point Likert scale (1 = completely disagree to 7 = completely agree), participants indicated their agreement with six statements about why AC is difficult (e.g., AC is difficult because I am not committed to it; because I am too tired) [47]. A summed score was computed for the six items, and was reliable (α = 0.84). 

#### 2.2.7. Coworker and Spouse active commuting Behavior

Using a 5-point Likert scale (1 = strongly disagree to 5 = strongly agree) participants responded to a question about their coworkers’ AC behavior: “*Most of my coworkers walk or bike to/from work*”. Participants also reported the number of times/week their spouse walked, biked or used public transportation to/from work. The sum of walking and bicycle trips was summed as spouse AC.

#### 2.2.8. Worksite

Participants indicated how much they perceived their employer supported AC with one item using a 5-point Likert scale (1 = strongly disagree to 5 = strongly agree). Respondents reported on their employer’s size and indicated (yes/no) number of employer supports for AC (e.g., incentives offered for AC, events related to AC, flexible work hours, bike storage policies, bicycle parking, locker rooms, flexible dress code), which were summed. Perceived problems for parking at work was assessed with three items about a lack of availability, high cost, and difficulty of parking with the same 1–5 Likert scale (greater score = more work parking problems).

#### 2.2.9. Community 

Participants reported on the availability of three supports for bicycling in their community (yes/no) (*i.e.*, bike racks on buses, covered bike parking, “*share the road*” signs), which were summed. Perceived support for walking and biking was assessed using a 5-point Likert scale (1 = strongly disagree to 5 = strongly agree), asking participants to indicate their agreement with five statements (e.g., town/city support for pedestrian or bicyclists issues, seeing others in their community walking or biking, and maintenance of sidewalks or bike lanes). Perceived community support was calculated by summing the scores. Perceived pedestrian and bike friendliness were assessed separately; with individuals rating their community using a 1 (the area is not pedestrian/bicycle friendly at all) to 5 (the area is very pedestrian/bicycle friendly) scale. Participants indicated how long they believed it would take them to walk or bike to work, which was dichotomized as ≤20 min and greater than 20 min.

#### 2.2.10. Environmental Barriers

With a 5-point Likert scale (1 = strongly disagree to 5 = strongly agree), participants indicated how perceived environmental barriers kept them from walking or biking to work. Items included: a lack of on-street bike lanes, lack of off-street walking/biking paths, lack of sidewalks, speed and volume of traffic along route, perceived crime along route, difficult terrain, and bad weather.

### 2.3. Analyses

Basic descriptive statistics and frequencies were used to describe the sample. *t*-test compared differences between public transit riders (PTR) and non-public transit riders (non-PTR). *t*-test and analyses of variances (ANOVAs) were used to examine differences between groups. Where necessary Tukey *post hoc* tests were used with the ANOVAs to examine significant differences between groups. Number of AC trips/week was dichotomized as 0 trips/more than one trip and binary logistic regression analyses were conducted with public transit travel as a constant variable to examine the effect of public transit use on AC. A complete logistic regression model was also conducted to examine factors that influence AC behavior. Significantly associated variables were included after accounting for colinearity, with the final model including individual, interpersonal, worksite, community and environmental variables. All analyses were performed using IBM SPSS 20.0 (Armonk, NY, USA) and significance levels were set at *p* < 0.05.

## 3. Results

Summary statistics of sample broken down by public transit use are shown in Table 1. Non-public transit riders (*n* = 596) reported driving to work 8.36 ± 3.37 times/week and actively commuted 1.57 ± 3.37 times/week. 

This was significantly different than public transit riders who reported driving to work 2.71 ± 3.54 times/week (*t* = 18.28, *p* < 0.001) and actively commuting 4.26 ± 4.55 times/week (*t* = 8.16, *p* < 0.001). PTR reported 4.43 ± 3.51 trips/week on public transit. The average number of cars per household (2.98) was higher than the US national average for 2012 of 2.08 cars/household [48].

### 3.1. Differences between Public Transit Riders and Non-Public Transit Riders

PTR were more likely to be male, report a lower income, be non-White and employed part time compared with NPTR. Non public transit riders were more likely to be older, report more children in the household, and more cars in the household. PTRs reported greater self-efficacy for biking skills, and greater perceived behavioral control for active commuting. NPTR were more likely to report being employed for longer than two years, compared with PTR who were more likely to report a shorter employment time. Spouse AC behavior and public transit ridership, as well as coworker AC behavior, were also greater among PTR. NPTR were more likely report their occupation as K-12 education (31.8%), higher education/research (21.2%) or white collar professionals (16.8%) whereas PTR were more likely to report higher education/research (36.2%) and white collar professionals (28.9%) as their occupation.

PTR were more likely to work at a larger employer (more than 100 employees) and reported greater perceived parking problems compared with NPTR. Also, PTR were more likely to perceive employer support for AC, report a greater number of employer supports for AC and community supports for AC compared with NPTR. PTR were less likely to report a lack of on-street bike lanes, a lack of off-street walking/biking paths, lack of sidewalks, the speed and volume of traffic along route, and difficult terrain as barriers to AC compared with NPTR.

**Table 1:** Characteristics of the Sample (*n* = 748).

Variable	Non Public Transit Riders (*n* = 596)		Public Transit Riders (*n* = 152)	
	*n* (%)	Mean (SD)	*n* (%)	Mean (SD)
Individual variables				
Age ^***^		43.30 (11.11)		39.54 (12.00)
Sex ^**^				
Male	184 (34.2)		65 (46.4)	
Female	354 (65.8)		75 (53.6)	
Marital Status (% Married/partnered)				
Married/partnered	423 (74.9)		101 (70.1)	
Single, divorced, widowed	142 (25.1)		43 (29.9)	
Race/ethnicity ^*^				
Non-Hispanic White	493 (92.5)		119 (85.6)	
Non-Hispanic Black	15 (2.8)		6 (4.3)	
All other racial/ethnic groups	25 (4.7)		14 (10.1)	
Number of children ^*^		0.56 (0.85)		0.37 (0.74)
Income level ^*^				
<$30 K/year	27 (5.2)		13 (9.5)	
$30–60 K/year	148 (28.6)		48 (35.0)	
>$60 K/year	343 (66.2)		76 (55.5)	
Education level				
High school graduate, some college	73 (13.6)		18 (12.9)	
College degree or higher	463 (86.4)		122 (87.1)	
Body Mass Index (kg/m^2^)		25.75 (5.45)		25.88 (5.30)
Number of reported chronic diseases		0.71 (1.07)		0.76 (1.07)
Number of cars in the household ^***^		2.98 (0.82)		2.29 (0.95)
Employment level (% employed full time) ^*^				
Employed full time	557 (93.9)		135 (88.8)	
employed part time	36 (6.1)		17 (11.2)	
Employment category ^*^				
Health/medical	43 (7.2)		9 (5.9)	
Administrative/clerical	48 (8.1)		17 (11.2)	
Education K-12	189 (31.8)		2 (1.3)	
Higher education/research	126 (21.2)		55 (36.2)	
Government/civil service	70 (11.8)		17 (11.2)	
Blue collar	18 (3.0)		8 (5.3)	
White collar	100 (16.8)		44 (28.9)	
Employment length ^***^				
less than 2 years	109 (18.4)		57 (37.7)	
2 years or more	483 (81.6)		94 (62.3)	
Self-efficacy for bicycling skills (range:1–4) ^**^		2.87 (1.07)		3.23 (1.04)
AC behavioral beliefs score (range:13–91)		69.41 (11.01)		69.74 (12.35)
Perceived behavioral control for AC (range: 7–42) ^***^		20.49 (7.97)		26.89 (8.99)
Interpersonal variables				
Spouse AC (times/week) ^**^		0.65 (2.25)		1.21 (3.07)
Spouse PT travel (times/week) ^***^		0.20 (1.23)		1.09 (2.84)
Perceived coworker AC (range:1–5) ^*^		1.53 (0.74)		1.82 (1.02)
Worksite variables				
Employer size ^*^				
1–100 employees	326 (54.9)		67 (44.4)	
more than 100 employees	268 (45.1)		84 (55.6)	
Number of employer supports for AC (range: 0–7) ^***^		2.37 (1.69)		2.96 (1.63)
Perceived employer support for AC (range: 1–5) ^**^		2.66 (1.33)		2.99 (1.23)
Perceived parking problems (range: 5–15) ^***^		6.20 (3.35)		8.69 (3.29)
Community variables				
Number of community supports for AC (range: 0–3) ^***^		1.46 (0.91)		1.94 (0.73)
Perceived community support for AC (range: 5–25)		16.82 (4.79)		17.12 (4.31)
Perceived pedestrian friendliness for AC (range: 1–5)		3.51 (1.24)		3.67 (1.23)
Perceive bicycle friendliness for AC (range: 1–5)		3.29 (1.24)		3.39 (1.14)
Perceived distance to work				
Less than 20 min bike ride	210 (37.7)		52 (35.4)	
Greater than 20 min bike ride	347 (62.3)		95 (64.6)	
Less than 20 min walk	81 (14.8)		14 (10.0)	
Greater than 20 min walk	467 (85.2)		126 (90.0)	
Environment variables (range 1–5)				
Lack of on street bike lanes ^***^		2.90 (1.53)		2.17 (1.30)
Lack of off street walking/biking paths ^***^		2.96 (1.55)		2.25 (1.40)
Lack of sidewalks ^***^		2.87 (1.57)		2.14 (1.43)
Speed and volume of traffic along route ^***^		3.27 (1.51)		2.81 (1.45)
Perceived crime along route		2.04 (1.35)		2.14 (1.34)
Difficult terrain ^*^		2.81 (1.46)		2.50 (1.37)
Bad weather		3.53 (1.34)		3.36 (1.37)

Note: AC: active commuting, PTR: public transit rider, NPTR: non-public transit rider, a significant difference between PTR and NPTR at the ^*^ *p* < 0.05, ^**^ *p* < 0.01, ^***^ *p* < 0.001 level.

### 3.2. Public Transit Riders’ Distances and Habits

Among PTRs, the following reported distances were observed: 59.6% (*n* = 99) report less than 0.5 miles total to/from their transit stop, 26.5% (*n* = 44) reported 0.5–1 miles to/from their transit stop and 13.9% (*n* = 23) reported more than 1 mile to/from their transit stop. Among transit riders, 78.1% (*n* = 125) never bring a bike on the transit vehicle, 16.9% (*n* = 27) report occasionally bringing a bike on public transit and 1.9% (*n* = 3) report sometimes or often doing so. Riders did not report disembarking early to walk or bike further; 60.1% (*n* = 95) report never getting off public transit early to walk/bike more, 25.3% (*n* = 40) reported occasionally doing this, and 14.6% (*n* = 23) reported doing this sometimes or often.

### 3.3. Factors Associated with Being an Active Commuter

The odds ratios showing the likelihood of being an active commuter with respect to individual variables while simultaneously controlling for public transit ridership are shown in Table 2. The results were as expected based on previous work and intuition. Having a younger age, having fewer children, a lower BMI, fewer cars and fewer chronic diseases were all associated with being an active commuter. Men were more likely to be an active commuter compared with women. 

**Table 2:** Odd ratios for being an Active Commuter, Controlling for Public Transit Use (*n* = 748).

Variable	Association with AC		
	OR	CI	*p*
Individual variables			
Age	0.94	0.93–0.96	<0.001
Sex			
Male (referent)	1		
Female	0.52	0.36–0.74	<0.001
Marital status			
Not married (referent)	1		
Married	0.6	0.42–0.87	0.007
Number of children	0.7	0.55–0.88	0.002
Income level			
<$30 K/year	1		
$30–60 K/year	0.58	0.28–1.19	0.14
>$60 K/year	0.46	0.23–0.92	0.03
Race/ethnicity			
Non-Hispanic White (referent)	1		
Non-Hispanic Black	0.3	0.08–1.08	0.06
All other racial/ethnic groups	2.61	1.30–5.23	0.007
Employment length			
less than 2 years	1		
2 years or more	0.61	0.42–0.90	0.01
Body Mass Index (kg/m^2^)	0.91	0.87–0.94	<0.001
Number of reported chronic diseases	0.73	0.61–0.88	0.001
Number of cars in the household	0.46	0.37–0.58	<0.001
Self-efficacy for bicycling skills	1.99	1.63–2.44	<0.001
AC behavioral beliefs score	1.04	1.02–1.06	<0.001
Perceived behavioral control for AC	1.22	1.18–1.27	<0.001
Interpersonal variables			
Spouse AC (times/week)	1.34	1.23–1.46	<0.001
Spouse PT travel (times/week)	1.06	0.97–1.16	0.22
Perceived coworker AC	1.8	1.46–2.22	<0.001
Worksite variables			
Employer size			
1–100 employees (referent)	1		
more than 100 employees	0.7	0.50–0.98	0.04
Number of employer supports for AC	1.22	1.10–1.34	<0.001
Perceived employer support for AC	1.43	1.25–1.63	<0.001
Perceived parking problems	1.07	1.02–1.13	0.004
Community variables			
Perceived distance to work			
Less than 20 min bike ride	3.6	2.26–5.73	<0.001
Greater than 20 min bike ride (referent)	1		
Less than 20 min walk	7.3	5.31–11.79	<0.001
Greater than 20 min walk (referent)	1		
Number of community supports for AC	0.26	0.17–0.90	<0.001
Perceived community support for AC	1.03	0.99–1.07	0.08
Perceived pedestrian friendliness for AC	1.43	1.23–1.66	<0.001
Perceive bicycle friendliness for AC	1.34	1.16–1.56	<0.001
Environment variables			
Lack of on street bike lanes	0.64	0.55–0.74	<0.001
Lack of off street walking/biking paths	0.6	0.52–0.69	<0.001
Lack of sidewalks	0.63	0.54–0.72	<0.001
Speed and volume of traffic along route	0.65	0.57–0.74	<0.001
Perceived crime along route	0.74	0.63–0.87	<0.001
Difficult terrain	0.62	0.54–0.73	<0.001
Bad weather	0.82	0.72–0.94	0.004

Note: AC: active commuting, PT: public transit.

Individuals who were married, employed at their current job for more than 2 years, and those reporting a household income of greater than $60,000/year were less likely to report AC, and those falling into “*other*” racial/ethnic groups were more likely to be active commuters. As expected, those with greater self-efficacy for biking, positive beliefs about AC and perceived behavioral control were more likely to be active commuters. Spouse and coworker AC participation was associated with being an active commuter, along with greater worksite support and more parking problems. Those working for a larger company (>100 employees) were less likely to be active commuters when controlling for PT use. Reporting fewer community supports for AC was associated with being an active commuter, and reporting a more bike and pedestrian-friendly community was also associated with AC. Those reporting a shorter walk and bike time were more likely to be active commuters. Those reporting fewer environmental barriers (lack of on-street bike lanes, off-street walking/bike paths or sidewalks, traffic, crime, difficult terrain or bad weather) were more likely to be active commuters.

### 3.4. Predictors of Active Commuting

The logistic multivariate analysis for AC (refer to Table 3) produced a Nagelkerke *R*^2^ = 0.776, indicating a good fit to the data. Age, number of children and perceived distance to work were negative predictors while feelings of bad weather and lack of on-street bike lanes as a barrier to active commuting, perceived behavioral control and spouse AC were positive predictors. Public transit ridership had the largest odds ratio (OR = 12.29 95% CI = 3.59–42.10, *p* < 0.001) of all variables, meaning that PT use was the most significant predictor of AC behavior of all variables.

**Table 3:** Logistic regression for Active Commuting (yes/no) (*n* = 748).

Variable	Association with AC		
	OR	95% CI	*p*
Individual variables			
Age	0.94	0.89–0.98	0.008
Number of children	0.49	0.28–0.88	0.02
Sex			
Male (referent)	1		
Female	0.68	0.28–1.63	0.43
Race/ethnicity			
Non-Hispanic White (referent)	1		
Non-Hispanic Black	3.61	0.40–32.84	0.25
Other racial/ethnic group	2.23	0.38–12.94	0.37
Income level			
<$30 K/year (referent)	1		
$30–60 K/year	0.55	0.05–6.53	0.64
>$60 K/year	3.14	0.22–45.08	0.4
Marital status			
Not married	3.63	1.12–11.74	0.03
Married (referent)	1		
Number of cars in the household	1.28	0.68–2.40	0.44
Self-efficacy for bicycling skills	1.11	0.68–1.81	0.67
AC behavioral beliefs score	1.02	0.98–1.06	0.35
Perceived behavioral control for AC	1.25	1.15–1.36	<0.001
Public transit use			
NPTR (referrent)	1		
PTR	12.29	3.59–42.10	<0.001
Interpersonal variables			
Spouse AC (times/week)	1.53	1.19–1.96	0.001
Perceived coworker AC	1.45	0.81–2.60	0.21
Worksite variables			
Perceived employer support for AC	1.28	0.90–1.84	0.17
Community variables			
Perceived pedestrian friendliness for AC	1.17	0.83–1.64	0.37
Perceived bike time to work			
More than 20 min (referent)	1		
Less than 20 min	7.23	2.59–20.17	<0.001
Environment variables			
Lack of on street bike lanes	1.86	1.08–3.21	0.02
Lack of sidewalks	0.65	0.40–1.08	0.1
Speed and volume of traffic along route	0.59	0.37–0.95	0.03
Perceived crime along route	1.1	0.71–1.70	0.66
Difficult terrain	1.16	0.77–1.74	0.48
Bad weather	1.86	1.28–2.70	0.001

Note: OR: odds ratio, CI: confidence interval, AC: active commuting, PTR: public transit rider, NPTR: Non public transit rider.

## 4. Discussion and Conclusions

Although limited, this study helps to reveal a connection between public transportation use and active commuting to work. Previous studies have revealed that taking public transit is associated with increased amounts of walking activity [29,30], which is most likely due to the fact that public transit users must walk or bicycle to access the transit line. These earlier works also found that public transit users have a higher likelihood of walking to destinations near the home and work [35] and generally have more active lifestyles [24]. However, this study shows that public transit use (even as little as once a week) is also associated with significantly fewer trips to work by car and more trips by active modes, similar to findings with Danish adults [49]. This relationship was first touched upon by Lachapelle and Frank [26], which found a positive correlation between having a transit pass and walking to work. In addition to the aforementioned reasons, casual observation suggests that some public transit riders tend to forego transit and instead walk or bicycle to their destination if they arrive to the transit stop just after a vehicle leaves (*i.e.*, when they expect a very long wait for the transit vehicle). If this is the case, the two travel modes are more intrinsically connected than previously thought. 

Perhaps more interestingly, this study is the first to reveal that public transit riders also perceive environmental barriers to active commuting less strongly than non-public transit riders, have a higher perceived behavioral control for active commuting and have higher self-efficacy for bicycling. The cause and effect nature of these relationships are not very clear, especially considering that land use patterns that influence commuter behavior was not considered; e.g., it is unknown if a traveler perceives environmental factors as less of a barrier because he is a frequent public transit user or if a traveler became public transit user because he perceives these environmental factors as less of a barrier. Weather could also be a factor in that someone who would typically walk or bike to work may opt for public transit during poor weather rather than driving. Further work is necessary to tease out this causality, but the existence of this relationship is certainly interesting. 

Despite the lack of direction of causality, when controlling for these types of behavioral factors public transit ridership appears to be a predictor of active commuting behavior to work among individuals. This result reinforces the conjecture by Burke and Brown [33] that increases in physical activity through walking and bicycling can be achieved by promoting public transportation service. Measures designed to induce a mode shift from cars to public transit—such as increasing transit service or subsidizing transit fares—may not only increase mobility and reduce vehicular emissions, but should also have the added benefit of promoting walking and bicycling to work. Recent evidence has suggested that having a well-connected and complete public transit network can result in a reduction in cars/household for some, which is related to increases in active travel [50]. Since the health benefits of active commuting modes are well documented [7,8,9,11,12,13], this further highlights the connection between public transportation service and better population health. Therefore, public transportation might not only be a vital component to livable cities [51], but also a key component to healthier communities as well.

In addition to public transit use, the results also highlight some other key insights about active commuting. Even when controlling for public transit use, psychological factors such as perceived behavioral control for AC, self-efficacy for bicycling and AC behavioral beliefs are significantly correlated with increased odds of being an active commuter. Thus, behavioral strategies might be successful in promoting active commuting. For example, classes designed to improve skills and comfort with urban cycling should help to increase someone’s self-efficacy for cycling and result in an increased likelihood that they will commute by bicycle [52]. Van der Kloof *et al.* [53] used this approach to build cycling skills with a population of non-Western immigrant women in Amsterdam and showed promising results. Educational outreach that stresses importance of daily physical activity and how this can be achieved by using active travel modes may also help to motivate commuters to walk or cycle to work. These behavioral strategies should especially targeted to those living in dense urban areas, where commute trips are generally shorter, since those with shorter perceived travel times to work are generally more inclined to use active modes.

Factors at the workplace were also found to be important in the likelihood of being an active commuter, even when controlling for public transit use. Similar to Kaczynski and colleagues findings with a sample from the US on social and physical supports as a significant influence on AC [54], the current study noted the importance of these types of influences. Not only is the number of employer supports for active modes (such as flexible work hours, lockers/changing rooms, bicycle parking) significantly correlated with increased active commuting, but also just the perception of employer support and parking problems at work increased the likelihood of someone being an active commuter. Therefore, targeted policies at the workplace (e.g., charging for parking or providing monetary incentives to those who do not drive to work) can help motivate employees to switch to either public transit or active modes, in addition to better employee education about the facilities and incentives offered by the employer. 

Despite the significant findings, there are some limitations that should be noted. The cross-sectional design employed by this study limit our ability to make causation statements or temporal assumptions about how the factors are related to AC behavior. Our sampling strategy and use of a volunteer, convenience sample limit the generalizability as well as our ability to objectively assess land use factors. Along with our strategy is the low response rate; however, given that the survey was delivered electronically, our reported response rate is most likely overly conservative as many of the e-invitations to complete the survey could have been filtered into “*spam*”/junk mail boxes and were never viewed by the potential participants. One of the most significant limitations is our reliance on self-report measures for AC behavior, public transit use and all interpersonal, worksite, community and environmental factors. Additional investigations in this area should consider using alternative or more objective forms of measurement for mode of transport and possible influence on travel mode choice. We also note that we did not ask about how individuals travel to or from their transit stops, so we lack data on these possibly multi-modal trips. Furthermore, we only examined active transportation to work and are unable to ascertain the role of active travel in general for individuals. Additional studies should investigate how AC is related to overall active transportation. Lastly, another limitation of this work is the inability to control for land use patterns around commuters’ work and homes. These land use patterns could also significantly affect both public transit use and active commuting behavior and should be taken into account in future studies. 

Notwithstanding these limitations, this study sheds light on the complex relationship between AC and public transit use. Given the extraordinary potential health, environmental and economic benefits, further research is needed to investigate how AC and public transit use are related and how one may moderate another. This study provides a foundation for future population-level health strategies to address AC. Further studies are being planned to address the limitations of the current effort.

## References

[1] Physical Activity Guidelines Advisory Committee (2008). Physical Activity Guidelines Advisory Committee Report, 2008.

[2] Brownson R.C., Boehmer T.K., Luke D.A. (2005). Declining rates of physical activity in the united states: What are the contributors?. Annu. Rev. Public Health.

[3] Haskell W.L., Lee I.M., Pate R.R., Powell K.E., Blair S.N., Franklin B.A., Macera C.A., Heath G.W., Thompson P.D., Bauman A. (2007). Physical activity and public health: Updated recommendation for adults from the american college of sports medicine and the american heart association. Med. Sci. Sports Exerc..

[4] Centers for Disease Control Prevention U.S. Physical Activity Statistics: 2007 State Demographic Data Comparison. http://www.cdc.gov/physicalactivity/data/.

[5] Robert Wood Johnson Foundation (2010). F as in Fat: How Obesity Threatens America’s Future.

[6] Centers for Disease Control and Prevention An Explanation of U.S. Physical Activity Surveys. http://www.cdc.gov/physicalactivity/professionals/data/explanation.html.

[7] Pucher J., Buehler R., Bassett D.R., Dannenberg A.L. (2010). Walking and cycling to health: A comparative analysis of city, state, and international data. Am. J. Public Health.

[8] Lindstrom M. (2008). Means of transportation to work and overweight and obesity: A population-based study in southern sweden. Prev. Med..

[9] Sugiyama T., Merom D., Reeves M., Leslie E., Owen N. (2010). Habitual active transport moderates the association of TV viewing time with body mass index. J. Phys. Act, Health.

[10] Wanner M., Gotschi T., Martin-Diener E., Kahlmeier S., Martin B.W. (2012). Active transport, physical activity, and body weight in adults: A systematic review. Am. J. Prev. Med..

[11] Hamer M., Chida Y. (2008). Active commuting and cardiovascular risk: A meta-analytic review. Prev. Med..

[12] Andersen L.B., Schnohr P., Schroll M., Hein H.O. (2000). All-cause mortality associated with physical activity during leisure time, work, sports, and cycling to work. Arch. Intern. Med..

[13] Shephard R.J. (2008). Is active commuting the answer to population health?. Sports Med..

[14] Humphreys D.K., Goodman A., Ogilvie D. (2013). Associations between active commuting and physical and mental wellbeing. Prev. Med..

[15] Hansson E., Mattisson K., Bjork J., Ostergren P.O., Jakobsson K. (2011). Relationship between commuting and health outcomes in a cross-sectional population survey in southern sweden. BMC Public Health.

[16] Westman J., Johansson M., Olsson L.E., Martensson F., Friman M. (2013). Children's affective experience of every-day travel. J. Transp. Geogr..

[17] U.S. Department of Health and Human Services (USDHHS) Healthy People 2020. http://www.healthypeople.gov/2020/default.aspx.

[18] Zheng Y. (2008). The benefit of public transportation: Physical activity to reduce obesity and ecological footprint. Prev. Med..

[19] Morabia A., Mirer F.E., Amstislavski T.M., Eisl H.M., Werbe-Fuentes J., Gorczynski J., Goranson C., Wolff M.S., Markowitz S.B. (2010). Potential health impact of switching from car to public transportation when commuting to work. Am. J. Public Health.

[20] Maibach E., Steg L., Anable J. (2009). Promoting physical activity and reducing climate change: Opportunities to replace short car trips with active transportation. Prev. Med..

[21] U.S. Census Bureau (2011). Commuting in the United States: 2009.

[22] Alliance for Biking & Walking (2014). Bicycling and Walking in the United States 2014 Benchmarking Report.

[23] American Public Transportation Association (2012). 2012 Public Transportation Fact Book. Appendix A: Historical Tables.

[24] Besser L.M., Dannenberg A.L. (2005). Walking to public transit: Steps to help meet physical activity recommendations. Am. J. Prev. Med..

[25] Lachapelle U. (2014). Walk, bicycle and transit trips of transit dependent and choice riders in the NHTS 2009. J. Phys. Act. Health.

[26] Lachapelle U., Frank L.D. (2009). Transit and health: Mode of transport, employer-sponsored public transit pass programs, and physical activity. J. Public Health Policy.

[27] Chaix B., Kestens Y., Duncan S., Merrien C., Thierry B., Pannier B., Brondeel R., Lewin A., Karusisi N., Perchoux C. (2014). Active transportation and public transportation use to achieve physical activity recommendations? A combined gps, accelerometer, and mobility survey study. Int. J. Behav. Nutr. Phys. ACT..

[28] Rissel C., Curac N., Greenaway M., Bauman A. (2012). Physical activity associated with public transport use—A review and modelling of potential benefits. Int. J. Environ. Res. Public Health.

[29] Edwards R.D. (2008). Public transit, obesity, and medical costs: Assessing the magnitudes. Prev. Med..

[30] MacDonald J.M., Stokes R.J., Cohen D.A., Kofner A., Ridgeway G.K. (2010). The effect of light rail transit on body mass index and physical activity. Am. J. Prev. Med..

[31] Wener R.E., Evans G.W. (2007). A morning stroll: Levels of physical activity in car and mass transit commuting. Environ. Behav..

[32] Litman T. (2014). Short and Sweet. Analysis of Shorter Trips Using National Personal Travel Survey Data.

[33] Litman T. (2013). Transportation and public health. Annu. Rev. Public Health.

[34] Villanueva K., Giles-Corti B., McCormack G. (2008). Achieving 10,000 steps: A comparison of public transport users and drivers in a university setting. Prev. Med..

[35] Lachapelle U., Frank L., Saelens B.E., Sallis J.F., Conway T.L. (2011). Commuting by public transit and physical activity: Where you live, where you work, and how you get there. J. Phys. Act. Health.

[36] Stokes R.J., MacDonald J., Ridgeway G. (2008). Estimating the effects of light rail transit on health care costs. Health Place.

[37] Rabi A., de Nazelle A. (2013). Benefits of shift from car to active transport. Transp. Policy.

[38] Baranowski T., Anderson C., Carmack C. (1998). Mediating variable framework in physical activity interventions. How are we doing? How might we do better?. Am. J. Prev. Med..

[39] De Bruijn G.J., Kremers S.P., Singh A., van den Putte B., van Mechelen W. (2009). Adult active transportation: Adding habit strength to the theory of planned behavior. Am. J. Prev. Med..

[40] Chaney R.A., Bernard A.L., Wilson B.R. (2013). Characterizing active transportation behavior among college students using the theory of planned behavior. Int. Q. Community Health Educ..

[41] Murtagh S., Rowe D.A., Elliott M.A., McMinn D., Nelson N.M. (2012). Predicting active school travel: The role of planned behavior and habit strength. Int. J. Behav. Nutr. Phys. Act..

[42] Troped P.J., Saunders R.P., Pate R.R., Reininger B., Addy C.L. (2003). Correlates of recreational and transportation physical activity among adults in a new england community. Prev. Med..

[43] Panter J.R., Jones A. (2010). Attitudes and the environment as determinants of active travel in adults: What do and don’t we know?. J. Phys. Act. Health.

[44] Panter J.R., Jones A.P., van Sluijs E.M., Griffin S.J., Wareham N.J. (2011). Environmental and psychological correlates of older adult’s active commuting. Med. Sci. Sports Exerc..

[45] Ajzen I. (1991). The theory of planned behavior. Organ. Behav. Hum. Decis. Process..

[46] Bandura A. (1986). Social Foundations of Thought and Action: A Social Cognitive Theory.

[47] Conn V.S., Tripp-Reimer T., Maas M.L. (2003). Older women and exercise: Theory of planned behavior beliefs. Public Health Nurs..

[48] Davis S.C., Diegel S.W., Boundy R.G. (2014). Chapter 8. Household vehicles and characteristics. Transportation Energy Data Book.

[49] Djurhuus S., Hansen H.S., Aadahl M., Glumer C. (2014). The association between access to public transportation and self-reported active commuting. Int. J. Environ. Res. Public Health.

[50] Transit Coopertive Research Program (2010). Current Practices in Greenhouse Gas Emissions. Savings from Transit.

[51] Vuchic V.R. (1999). Transportation for Livable Cities.

[52] Yang L., Sahlqvist S., McMinn A., Griffin S.J., Ogilvie D. (2010). Interventions to promote cycling: Systematic review. BMJ.

[53] van der Kloof A., Bastiaanssen J., Martens K. (2014). Bicycle lessons, activity participation and empowerment. Case Stud. Transp. Policy.

[54] Kaczynski A.T., Bopp M.J., Wittman P. (2010). Association of workplace supports with active commuting. Prev. Chronic Dis..

